# Graphical models for inferring single molecule dynamics

**DOI:** 10.1186/1471-2105-11-S8-S2

**Published:** 2010-10-26

**Authors:** Jonathan E Bronson, Jake M Hofman, Jingyi Fei, Ruben L Gonzalez, Chris H Wiggins

**Affiliations:** 1 Department of Chemistry, Columbia University, New York, NY 10027, USA; 2 Yahoo! Research, 111 West 40th St., New York, NY 10018, USA; 3 Department of Applied Physics and Applied Mathematics, Columbia University, New York, NY 10027, USA

## Abstract

**Background:**

The recent explosion of experimental techniques in single molecule biophysics has generated a variety of novel time series data requiring equally novel computational tools for analysis and inference. This article describes in general terms how graphical modeling may be used to learn from biophysical time series data using the variational Bayesian expectation maximization algorithm (VBEM). The discussion is illustrated by the example of single-molecule fluorescence resonance energy transfer (smFRET)* versus* time data, where the smFRET time series is modeled as a hidden Markov model (HMM) with Gaussian observables. A detailed description of smFRET is provided as well.

**Results:**

The VBEM algorithm returns the model’s evidence and an approximating posterior parameter distribution given the data. The former provides a metric for model selection via maximum evidence (ME), and the latter a description of the model’s parameters learned from the data. ME/VBEM provide several advantages over the more commonly used approach of maximum likelihood (ML) optimized by the expectation maximization (EM) algorithm, the most important being a natural form of model selection and a well-posed (non-divergent) optimization problem.

**Conclusions:**

The results demonstrate the utility of graphical modeling for inference of dynamic processes in single molecule biophysics.

## Background

**Figure 1:**
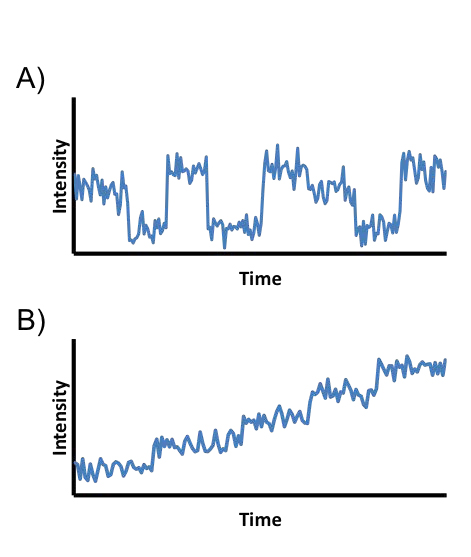
**Examples of types of commonly encountered biophysical time series data.** (A) A time series for a molecule transitioning between a series of locally stable conformations. Such data often arise, for example, when studying protein domain movements or the dynamics of polymers tethered to a surface. (B) A time series for a molecule undergoing a stepping process. Such data often arise, for example, when studying proteins with unidirectional movements, e.g., helicases and motor proteins.

Single-molecule techniques allow biophysicists to probe the dynamics of proteins, nucleic acids, and other biological macromolecules with unprecedented resolution [1-3]. It is now possible to observe viruses pack DNA into capsids [4], helicases unzip nucleic acids [5], motor proteins walk on biopolymers [6], and ribosome domains undergo structural rearrangements during translation [7]. These data are acquired by recording the fluorescent output or forces generated from, for example, biomolecules tethered onto microscope slides [8]; walking on biopolymers [9]; diffusing in hydrodynamic flow cells [10]; or pulled by optical [11] or magnetic [12] tweezers. Often the molecules studied move through a series of locally stable molecular conformations or positions (generically termed states) and give rise to data of the type shown in Fig. 1. From these data, the experimentalist wishes to learn a model describing the number of states occupied by the molecule and the transition rates between states. Although the myriad experimental techniques available have much in common, the data they generate often differ enough to require unique models.

For example, some of these models will involve conversion of chemical to mechanical energy, or motion associated with diffusion, or motion associated with transitions between distinct configurational states. Modeling the data, then, typically involves introducing several variables — some of which are observed, others of which are latent or “hidden”; some of which are real-valued coordinates, others of which are discrete states — and specifying algebraically how they are related. Such algebraic relations among a few variables are typical in physical modeling (e.g., the stochastic motion of a random walker, or the assumption of additive, independent, normally distributed errors typical in regression); models involving multiple conditionally-dependent observations or hidden variables with more structured noise behavior are less common. Implicitly, each equation of motion or of constraint specifies which variables are conditionally dependent and which are conditionally independent. Graphical modeling, which begins with charting these dependencies among a set of nodes, with edges corresponding to the conditional probabilities which must be algebraically specified (i.e., the typical elements of a physical model) organizes this process and facilitates basing inference on such models [13-15].

Here we explore the application of a specific subset of GMs to biophysical time series data using a specific algorithmic approach for inference: the directed GM and the variational Bayesian expectation maximization algorithm (VBEM). After discussing the theoretical basis and practical advantages of this general approach to modeling biophysical time series data, we apply the method to the problem of inference given single molecule fluorescence resonance energy transfer (smFRET) time series data. We emphasize the process and caveats of modeling smFRET data with a GM and demonstrate the most helpful features of VBEM for this type of time series inference.

### Graphical models

GMs are a flexible inference framework based on factorizing a (high-dimensional) multivariate joint distribution into (lower-dimensional) conditionals and marginals [13-15]. In a GM, the nodes of the graph represent either observable variables (data, denoted by filled circles), latent variables (hidden states, denoted by open circles), or fixed parameters (denoted by dots). Directed edges between nodes represent conditional probabilities. For example, the three-node graphical model* X* → *Y* → *Z* implies that the joint distribution* p*(*Z,Y*, *X*) ≡* p*(*Z|Y*, *X*)*p*(*Y*|*X*)*p*(*X*) can be further factorized as* p*(*Z*|*Y*)*p*(*Y*|*X*)*p*(*X*). Data with a temporal component are modeled by connecting arrows from variables at earlier time steps to variables at later time steps. In many graphical models, the number of observed and latent variables grows with the size of the data set under consideration. To avoid clutter, these variables are written once and placed in a box, often called a “plate”, labeled with the number of times the variables are repeated [15]. This manuscript will denote hidden variables by *z* and observed data by *d*. Parameters which are vectors will be denoted as such by overhead arrows.

**Figure 2:**
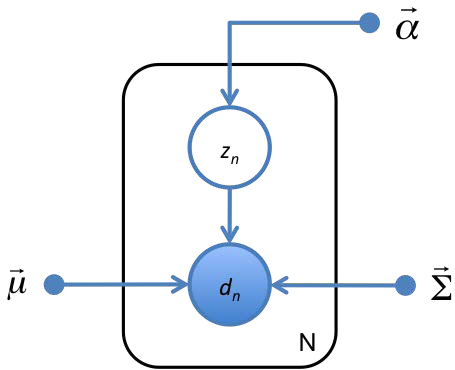
**A GM for the problem of learning genders of boys and girls from a table of their heights and weights.** The gender of the *n^th^* child is denoted *z_n_*. The 2-dimensional vector of the child’s height and weight is denoted* d_n_* The mean hight and weight for each gender, variances of height and weight for each gender, and probability of belonging to each gender are denoted by , , and , respectively. Observed variables are represented by open circles, hidden variables are represented by filled circles, and fixed parameters are represented by dots. To avoid drawing nodes for all *N* hidden and observed variables, the variables are shown once and placed inside a plate which denotes the number or repetitions in the lower right corner. This GM specifies the conditional factorization of  shown in Eq. 1.

As an example of a simple GM, imagine trying to learn the number of boys and girls in an elementary school class of *N* students from a table of their heights and weights. Here the hidden variable is gender and the observed variable, (height, weight), is a random variable conditionally dependent on the hidden variable. The resulting GM is shown in Fig. 2, with the parameters of* p(gender)* denoted by  and the parameters* p*(*height, weight|gender*) denoted by  and . The expression for the probability of the observed data ({*d*_1_,…,*d_N_*} = **D**) and latent genders ({*z*_1_,…,*z_N_*} = **Z**) is uniquely specified by the graph and the factorization it implies:

	(1)

In such a simple case it is straighforward to arrive at the expression in Eq. 1 without the use of a GM, but such a chart makes this factorization far more obvious and interpretable.

### Inference of GMs

In some contexts, one wishes to learn the probability of the hidden states given the observed data,  where  denotes the parameters of the model and* K* denotes the number of allowed values of the latent variables (i.e. number of hidden states). If  is known then efficient inference of  can be performed on any loop-free graph with discrete latent states using the* sum-product* algorithm [16], or, if only the most probable values of **Z** are needed, using the closely related* max-sum* algorithm [17]. A loop in a graph occurs when multiple pathways connect two variables, which is unlikely in a graph modeling time series data. Inference for models with continuous latent variables is discussed in [18,19]. For most time series inference problems in biophysics, both **Z** and  are unknown. In these cases, a criterion for choosing a best estimate of  and an optimization algorithm to find this estimate are needed.

#### *Inference via maximum likelihood*

Estimating  is most commonly accomplished using the* maximum likelihood* (ML) method, which estimates  as

 (2)

The probability  is known as the* likelihood.* The expectation maximization (EM) algorithm can be used to estimate [20]. In EM, an initial guess for  is used to calculate . The  learned is then used to calculate a new guess for . The algorithm iterates until convergence, and is guaranteed to converge to a local optimum. The EM algorithm should be run with multiple initializations of , often called “random restarts”, to increase the probability of finding the globally optimal .

ML solved via EM is a generally effective method to perform inference however, it has two prominent shortcomings [14,15]:

**Model selection:** The first limitation of ML is that it has no form of model selection: the likelihood monotonically increases with the addition of more model parameters. This problem of fitting too many states to the data (overfitting) is highly undesirable for biophysical time series data, where learning the correct *K* for the data is often an experimental objective.

**Ill-posedness** The second problem with ML occurs only in the case of a model with multiple hidden states and a continuous observable (a case which includes the majority of biophysical time series data, including the smFRET data we will consider here). If the mean of one hidden state approaches the position of a data point and the variance of that state approaches zero, the contribution of that datum to the likelihood will diverge. When this happens, the likelihood will be infinite regardless of how poorly the rest of the data are modeled, provided the other states in the model have non-zero probabilities for the rest of the data. For such models, the ML method is ill-posed; poor parameters can still result in infinite likelihood.

In practical contexts, the second problem (divergent likelihood) can be avoided either by performing MAP estimation (maximizing the likelihood times a prior which penalizes small variance) or by ignoring solutions for which likelihood is diverging. That is, one does not actually maximize the likelihood. Model selection can then be argued for based on cross-validation or by penalizing likelihood with a term which monotonically increases with model complexity [15,21,22]. We consider, instead, an alternative optimization criterion which naturally avoids these problems.

#### *Inference via maximum evidence*

A Bayesian alternative to ML is to perform inference using the* maximum evidence* (ME) method. ME can be thought of as an extension of ML to the problem of model selection. Where ML asks which parameters maximize the probability of the data for a given model, ME asks which model, including nested models which differ only in *K*, makes the data most probable. According to ME, the model of correct complexity (K_*_) is

 (3)

The quantity  is called the evidence. Sometimes it is also referred to as the marginal likelihood, since unknown parameters are assigned probability distributions and marginalized (or summed out) over all possible settings. The evidence penalizes both models which underfit and models which overfit. The second expression in Eq. 3 follows readily from the sum rule of probability provided we are willing to model the parameters themselves as random variables. That is, we are willing to specify a distribution over parameters, . This distribution is called the “prior”, since it can be thought of as the probability of the parameters prior to seeing any data. The parameters for the distributions of the prior  are called *hyperparameters.* In addition to providing a method for model selection, by integrating over parameters to calculate the evidence rather than using a “best” point estimate of the parameters, ME avoids the ill-posedness problem associated with ML.

Although ME provides an approach to model selection, calculation of the evidence does not, on its own, provide an estimate for  The VBEM approach to estimating evidence does, however, provide a mechanism to estimate . VBEM can be thought of as an extension of EM for ME. Both the VBEM algorithm and considerations for choosing priors are discussed in Methods.

### smFRET

Before building a GM describing smFRET data, it is helpful to review briefly the experimental method. The experimental technique is based on the spectroscopic phenomenon that, if the emission spectrum of a polar chromophore (donor) overlaps with the absorption spectrum of another polar chromophore (acceptor), electromagnetic excitation of the donor can induce a transfer of energy to the acceptor via a non-radiative, dipole-dipole coupling process termed florescence resonance energy transfer (FRET) [23]. The transfer efficiency between donor and acceptor scales with the distance between molecules (r) as 1/*r*^6^, with FRET efficiencies most sensitive to r in the range of 1 − 10nm. Because of this extraordinary sensitivity to distance, FRET efficiency can serve as a molecular ruler, allowing an experimentalist to measure the separation between donor and acceptor by stimulating the donor with light and measuring emission intensities of both the donor (*I_D_*) and acceptor (*I_A_*) [24]. Usually a summary statistic called the “FRET ratio” is used to report on molecular distance rather than the “raw”, 2-channel {*I_A_*,*I_D_*} data, although inference of the raw 2-channel data is possible as well [25]. The FRET ratio is given by

  (4)

**Figure 3:**
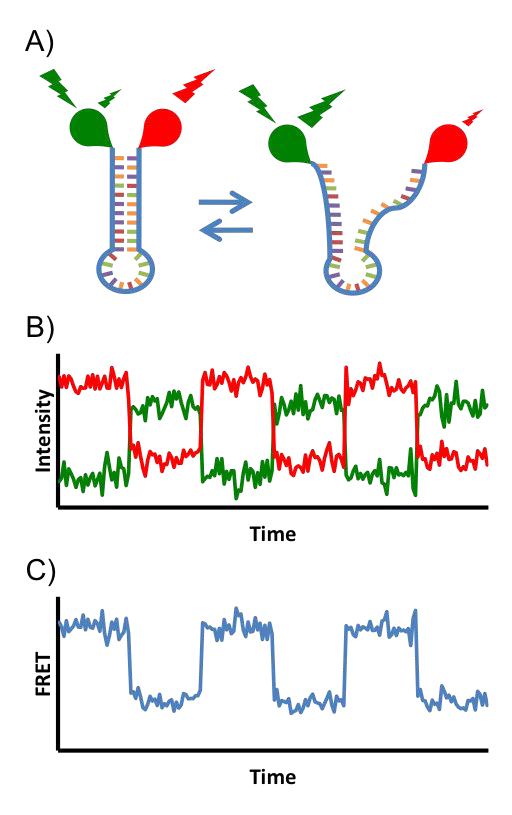
**(A) Cartoon of a smFRET experiment studying the zipping/unzipping of a DNA hairpin.** A FRET donor chromophore (green balloon) and acceptor chromophore (red balloon) are attached to the DNA. When the DNA is zipped (left), exciting the donor with green light causes the majority of energy to be transferred to the acceptor. The donor will fluoresce dimly and the acceptor will fluoresce brightly. When the DNA is unzipped, the probes are too far apart for efficient FRET. Exciting the donor in this conformation causes it to fluoresce brightly and the acceptor to fluoresce dimly. (B) The two channel (donor, acceptor) time series generated by the DNA as it transitions between zipped (bright red, dim green) and unzipped (dim red, bright green). (C) The 1D FRET transformation of the time series from B, calculated with Eq. 4. The closer the probes, the more intense the signal. Time series of this summary statistic are commonly used for analysis.

When the donor and acceptor are attached to an individual protein, nucleic acid, or other molecular complex, the FRET signal can be used to report on the dynamics of the molecule to which the donor and acceptor are attached (see Fig. 3). When the experiment is crafted to monitor individual molecules rather than ensembles of molecules, the process is termed single molecule FRET (smFRET). For many biological studies, such as the identification and characterization of the structural dynamics of a biomolecule, smFRET must be used rather than FRET; the majority of molecular dynamics cannot be observed from ensemble averages. Often the molecule of interest adopts a series of locally stable conformations during a smFRET time series. From these data, the experimentalist would like to learn (1) the number of locally stable conformations in the data (i.e. states) and (2) the transition rates between states. Although it is theoretically possible use the FRET signal to quantify the distance between parts of a molecule during a time series, there are usually too many variables affecting FRET efficiency for this to be practical [26]. Consequently, smFRET is usually used to extract quantitative information about kinetics (i.e. rate constants) but only qualitative information about distances.

The photophysics of FRET have been studied for over half a century, but the first smFRET experiments were only carried out about fifteen years ago [27]. The field has been growing exponentially since, and hundreds of smFRET papers are published annually [1]. Diverse topics such as protein folding [28], RNA structural dynamics [29], and DNA-protein interactions [30] have been investigated via smFRET. The size and complexity of smFRET experiments has grown substantially since the original smFRET publication. A modern smFRET experiment can generate thousands of time series to be analyzed [7].

## Results and discussion

### smFRET as a graphical model

**Figure 4:**
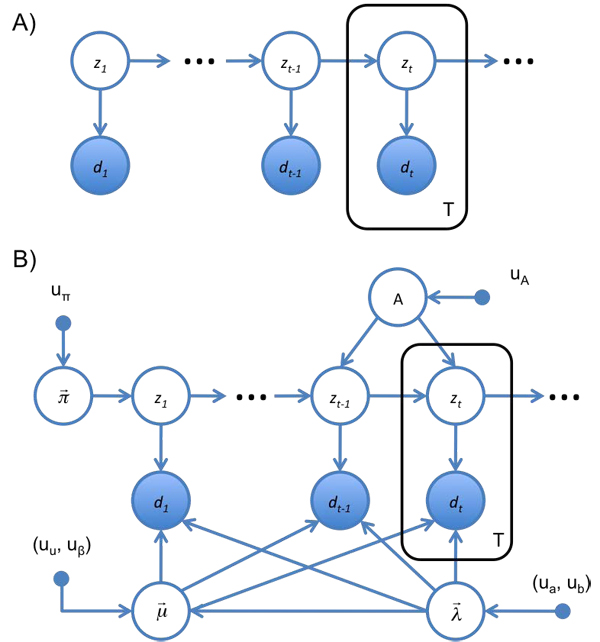
**(A) The HMM as a GM.** At each time step, *t*, the system occupies a hidden state, *z_t_* and produces an observable emission, *d_t_*, drawn from *p*(*d_t_*|*z_t_*). In turn, *z_t_* is drawn from *p*(*z_t_*|*z_t_*_−1_). (B) Complete GM for the HMM used to describe smFRET data in this work. Following the Bayesian treatment of probability, all unknown parameters are treated as hidden variables, and represented as open circles. Emissions are assumed to be Gaussian, with mean  and precision . Transition rates are multinomial, with probabilities given by *A*. The probability of initially occupying each hidden state is multinomial as well, with probabilities given by . Equations for these distributions are described in the text below Eq. 5. This GM specifies the conditional factorization of  shown in Eq. 6.

A model of the smFRET time series for a molecule transitioning between a series of locally stable conformations should capture several important aspects of the process [31]. The observable smFRET signal is a function of the hidden conformation of the molecule. The noise of each smFRET state can be assumed to be Gaussian, and the hidden conformations are assumed to be discrete and finite in number. The probability of transitioning to a new molecular conformation should be a function of the current conformation of the molecule (*e.g.,* the DNA in Fig. 3 is more likely to be zipped at time *t* + 1 if it is zipped at time *t*). The CCD cameras commonly used in smFRET experiments naturally bin the data temporally, so it is convenient to work with a model where time is discrete. The GM expressing these features is called a hidden Markov model (HMM) and is shown in Fig. 4A. From the graph, it can be seen that the probability of the observed and latent variables factorizes as

  (5)

Here,  must include parameters for the probability that the time series begins in each state (*p*(*z*_1_ = *k*) ≡ π*_k_*); parameters for transition probabilities between states (*p*(*z_t_*_+1_ =* k|z_t_* =* j*) ≡* a_jk_*); and parameters for the noise of the emissions of each state (*p*(*d_t_*|*z_t_* = *k*) =* N*(*d_t_|µ_k_,*λ*_k_*), where *µ_k_* and λ*_k_* are the mean and precision of the Gaussian). It is necessary to model *p*(*z*_1_) separately from all other transition probabilities since it is the only hidden state probability which does not depend on *z_t_*_−1_. The *a_jk_* are commonly represented as a matrix, *A*, called a transition matrix. The probability the time series begins in the* k^th^* state and transition probabilities between states are drawn from multinomial distributions defined by  and the rows of *A*, respectively. The GM for this HMM is shown in Fig. 4B. From the GM it can be seen that

  (6)

For a time series of length T where each latent variable can take on K states, a brute summation over all possible states requires* O*(*K^T^*) calculations. By exploiting efficiencies in the GM and using the sum-product algorithm, this summation can be performed using* O*(*K*^2^*T*) calculations (which can be seen by noting that the latent state probabilities in Eq. 6 factorize into *p*(*z_t_*|*z_t_*_−1_*, A, K*), where each of the* T* latent states has* K*^2^ possible combinations of states). The sum-product algorithm applied to the HMM is called the forward-backward algorithm or the Baum-Welch algorithm [32], and the most probable trajectory is called the Viterbi path [33].

There are several assumptions of this model which should be considered. First, although it is common to assume the noise of smFRET states is Gaussian, the assumption does not have a theoretical justification (and since FRET intensities can only be on the interval (0,1), and the Gaussian distribution has suport (−∞, ∞), the data cannot be truly Gaussian). Despite this caveat, several groups have successfully modeled smFRET the data as having Gaussian states [25,34,35]. We note that other distributions have been considered as well [36].

Second, the HMM assumes that the molecule instantly switches between hidden states. If the time it takes the molecule to transition between conformations is on the same (or similar) order of magnitude as the time it spends within a conformation, the HMM is not an appropriate model for the process and a different GM will be needed. For many molecular processes, such as protein domain rearrangements, the molecule transitions between conformations orders of magnitude faster than it remains in a conformation and the HMM can model the process well [37].

Third, the HMM is “memoryless” in the sense that, given its current state, the transition probabilities are independent of the past. It is still possible to model a molecule which sometimes transitions between states quickly and sometimes transitions between states slowly (if, for example, binding of another small molecule to the molecule being studied changes its transition rates [7]). This situation can be modeled using two latent states for each smFRET state. The two latent states will have the same emissions model parameters, but different transition rates.

### Illustration of the inference

**Figure 5:**
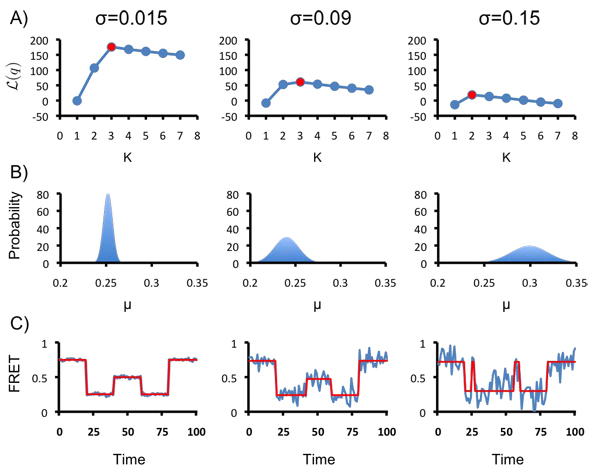
**(A) Model selection using ME.** Inference using 1 ≤* K* ≤ 7 hidden states was performed for each trace. The results with the highest *L*(*q*) are shown in red. (B) The posterior parameter distribution for the lowest-valued smFRET state inferred in each time series. The width of the posterior increases with the noise of the smFRET states, indicating lower confidence in the parameters learned from inference on noisier time series. (C) The idealized trajectories (red) inferred for each time series (blue) using the most probable parameters of the inference with the highest *L*(*q*).

A software package, vbFRET, implementing the VBEM algorithm for this HMM was written and described in [25], along with an assessment of the algorithm’s performance on real and synthetic data. An illustration of the method is shown here, demonstrating three of its most important abilities: the ability to perform model selection; the ability to learn posterior parameter distributions; and the ability to idealize a time series. These abilities are demonstrated on three synthetic *K* = 3 state time series, shown in Fig. 5C. The traces all have* µ* = {0.25,0.5, 0.75} and identical hidden state trajectories. The noise of each hidden state is* σ* = 0.015 for trace 1 (unrealistically noiseless),* σ* = 0.09 for trace 2 (a level of noise commonly encountered in experiments), and* σ* = 0.15 for trace 3 (unrealistically noisy).

Model selection: For each trace, *L*(*q*), the lower bound of the log(evidence), was calculated for 1 ≥ *K* ≥ 7. The results are shown in Fig. 5A, with the largest value of *L*(*q*) for each trace shown in red. For traces 1 and 2, *L*(*q*) peaks for K_*_ = 3, correctly inferring the complexity of the model. For trace 3, the noise of the system is too large, given the length of the trace, to infer three clearly resolved states. For this trace *L*(*q*) peaks at K_*_ = 2. This result illustrates an important consideration of evidence based model selection: states which are distinct in a generative model (or an experiment generating data) may not give rise to statistically significant states in the data generated. For example, two states which have identical means, variances, and transition rates would be statistically indistinguishable from a single state with those parameters. When states are resolvable, however, ME-based model selection is generally effective, as demonstrated in traces 1 and 2.

**Posterior distributions:** The ability to learn a complete posterior distribution for  provides more information than simply learning an estimate for , and is a feature unique to Bayesian statistics. The maximum of the distribution, denoted , can be used as an estimate of  (e.g., if idealized trajectories are needed). The subscript here differentiates it from the estimate in the absence of the prior, . The curvature of the distribution describes the certainty of the  estimate. As a demonstration, the posterior for the mean of the lowest smFRET state of each trace is shown in Fig. 5B. The X and Y axes are the same in all three plots, so the distributions can be compared. As expected, the lower the noise in the trace, the narrower the posterior distribution and the higher the confidence of the estimate for The estimate of* µ* for trace 3 is larger than in the other traces because K_*_ = 2; some the middle smFRET state data are misclassified as belonging to the low smFRET state.

**Idealized trajectories:** Idealized smFRET trajectories can be a useful visual aid to report on inference. They are also a necessity for some forms of post-processing commonly used at present, such as dwell-time analysis [7]. Idealized trajectories can be generated from the posterior learned from VBEM by using  to calculate the most probable hidden state trajectory (the Viterbi path) [33]. The idealized trajectories for each trace are shown in Fig. 5C. For traces 1 and 2, where K_*_ is correctly identified, the idealized trajectory captures the true hidden state trajectory perfectly. Because of the model selection and well-posedness of ME/VBEM the idealized trajectories learned with this method can be substantially more accurate than those learned by ML for some data sets [25].

## Conclusions

This manuscript demonstrates how graphical modeling, in conjunction with a detailed description of a biophysical process, can be used to model biophysical time series data effectively. The GM designed here is able to model smFRET data and learn both the number of states in the data and the posterior parameter values for those states. The ME/VBEM methodology used here offers several advantages over the more commonly used ML/EM inference approach, including intrinsic model selection and a well-posed optimization. All modeling assumptions are readily apparent from the GM. The GM framework with inference using ME/VBEM is highly flexible modeling approach which we anticipate will be applicable to a wide array of problems in biophysics.

## Methods

All code used in this manuscript is available open source at http://vbfret.sourceforge.net/.

### Variational Bayesian expectation maximization

Unfortunately, calculation of Eq. 3 requires a sum over all K settings for each of T extensive variables **Z** (where T is the length of the time series). Such a calculation is numerically intractable, even for reasonably small systems (*e.g.,* K=2, T=100) so an approximation to the evidence must be used. Several approximation methods exist, such as Monte Carlo techniques, for numerically approximating such sums [38]. The method we will consider here is VBEM.

One motivation for the VBEM algorithm is the following simple algebraic identity [15]. Since Bayesian analysis treats latent variables (**Z**) and unknown parameters  the same way this section will lump them both into **X** for notational simplicity. Let *q*(**X**) be any probability distribution over X. Then,

  (7)

 (8) 

  (9)

 (10) 

  (11)

Summations over the discrete components of **X** should be included in these equations, but are omitted for notational simplicity. The equality in Eq. 7 results from the requirement that *q*(**X**) be a normalized probability; Eq. 8 rewrites  in terms of a conditional probability; and Eq. 11 reinserts  for **X** and renames the two terms in Eq. 10 as *L*(*q*), the lower bound of the log(evidence), and the Kullback-Leibler divergence, respectively.

Using Jensen’s inequality, it can be shown that

*D_kL_* (*q*| |*p*) ≥ 0, (12)

with equality when* q* =* p.* Consequently,

  (13)

*i.e.,* exp(*L*(*q*)) is a lower bound on the model’s evidence. Eq. 12 implies that *L*(*q*) is maximized when  is equal to . As a corollary, from this it follows that* q*(*θ*) approximates , the* posterior* distribution of the parameters. Therefore, the optimization simultaneously performs model selection (by finding a *K* which maximizes ) and inference (by approximating ). 

The approach suggested by Eqs. 7–12 is to replace an intractable calculation with a tractable bound optimization. If  is in the exponential family and a conjugate prior is used, then the only assumption about  needed is that  (*i.e.,* it factorizes into a function of **Z** and a function of ) for the inference problem to be tractable using VBEM [39]. In addition, under these conditions  will have the same functional form as . The VBEM algorithm is similar to EM, but rather than iteratively using guesses for  to set **Z** and guesses for **Z** to set  the update equations iterate between [15,40]:

  (14)

 (15)

Here  denotes the expected value with respect to the subscripted distribution and  is a normalization constant. Whereas the  is a log of a sum/integral, the right hand sides of Eqs. 14 & 15 both involve the sum/integral of a log. This difference renders  intractable, yet Eqs. 14 & 15 tractable.

An interesting and potentially useful feature of the  learned from VBEM is that when *K* is chosen to be larger than the number of states supported by the data, the optimization leaves the extra states unpopulated. This propensity to leave unnecessary states unpopulated in the posterior, sometimes called “extinguishing”, is a second form of model selection intrinsic to VBEM, which is in addition to the model selection described by Eq. 3. An explanation for this behavior can be found in Chapter 3 of [15].

### Priors

Several considerations should go into choosing a prior. Choosing distributions which are conjugate to the parameters of the likelihood can greatly simplify inference [39]. Priors can be chosen to minimize their influence on the inference. Such priors are called “weak” or uninformative. Alternatively, priors can also be chosen to respect previously obtained experimental observations [40]. It is important to check that inference results do not heavily depend on the prior (*e.g.* doubling or halving hyperparameter values should not affect inference results).

The conjugate prior of a multinomial distribution is a Dirichlet distribution:  Expressed in terms of precision λ, rather than variance *σ*^2^ (where λ = 1/*σ*^2^), the conjugate prior for the mean and precision of a Gaussian is a Gaussian-Gamma distribution: .

Here, hyperparameters were set so as to give distributions consistent with experimental data and to influence the inference as weakly as possible:  and , for all values of *k*. Qualitatively, these hyperparameters specify probability distributions over the hidden states such that it is most probable that the hidden states are equally likely to be occupied and equally likely to be transitioned to. Quantitatively, they yield 〈µk〉= 0.5 and mode[*σ*] ≈ 0.08, consistent with experimental observation:

  (16)

### Data generation

Synthetic traces were generated in MATLAB using 1-D Gaussian noise for each hidden state and a manually determined hidden state trajectory. All traces were analyzed by vbFRET [25], using its default parameter settings, for 1 ≥* K* ≥ 7, with 25 random restarts for each value of* K.* The restart with the highest evidence was used to generate the data in Fig. 5. The posterior probability of is given by , where  are the hyperparameters of the posterior. The data in Fig. 5B were generated using this equation with λ*_k_* fixed at its most probable posterior value.

## Competing interests

The authors declare that they have no competing interests.

## Authors contributions

JEB contributed to the graphical modeling and smFRET inference. JMH contributed to the graphical modeling. JF and RLG contributed to the smFRET inference. CHW contributed to the graphical modeling and smFRET inference. JEB and CHW wrote the manuscript.
